# Acceptance and Commitment Therapy to manage pain and opioid use after major surgery: Preliminary outcomes from the Toronto General Hospital Transitional Pain Service

**DOI:** 10.1080/24740527.2017.1325317

**Published:** 2017-06-28

**Authors:** Muhammad Abid Azam, Aliza Z. Weinrib, Janice Montbriand, Lindsay C. Burns, Kayla McMillan, Hance Clarke, Joel Katz

**Affiliations:** a Pain Research Unit, Department of Anesthesia and Pain Management, Toronto General Hospital, Toronto, Ontario, Canada; b Department of Psychology, York University, Toronto, Ontario, Canada

**Keywords:** chronic postsurgical pain, persistent opioid use, multidisciplinary pain management, acceptance and commitment therapy, pain interference, pain catastrophizing, depression, anxiety, mindfulness, douleur post-chirurgicale chronique, interférence de la douleur, catastrophisation de la douleur, dépression, anxiété, pleine conscience

## Abstract

**Background**: Chronic postsurgical pain (CPSP) and associated long-term opioid use are major public health concerns.

**Aims**: The Toronto General Hospital Transitional Pain Service (TPS) is a multidisciplinary, hospital-integrated program developed to prevent and manage CPSP and support opioid tapering. This clinical practice–based study reports on preliminary outcomes of the TPS psychology program, which provides acceptance and commitment therapy (ACT) to patients at risk for CPSP and persistent opioid use.

**Methods**: Ninety-one patients received ACT, whereas 252 patients did not (no ACT group). Patient outcomes were compared for the two groups at first and last TPS visits. Pain, pain interference, sensitivity to pain traumatization, pain catastrophizing, anxiety, depression, and opioid use were analyzed using two-way (Group [ACT, no ACT] × Time [first, last visit]) analyses of variance (ANOVAs).

**Results**: Patients referred to ACT were more likely to report a mental health condition preoperatively (*P* < 0.001), had higher opioid use (*P* < 0.001) at the first postsurgical visit, and reported higher sensitivity to pain traumatization (*P* < 0.05) and anxiety (*P* < 0.05) than the no ACT group at both time points. Both groups showed reductions in pain, pain interference, pain catastrophizing, anxiety, and opioid use by the last TPS visit (*P* < 0.05). The ACT group demonstrated greater reductions in opioid use and pain interference and showed reductions in depressed mood (*P* = 0.001) by the end of treatment compared to the no ACT group.

**Conclusion**: Preliminary outcomes suggest that ACT was effective in reducing opioid use while pain interference and mood improved.

## RÉSUMÉ

**Contexte**: La douleur chronique post-chirurgicale (DCPC) et l’usage à long terme d’opioïdes qui y sont associées sont des préoccupations majeures en santé publique.

**Objectifs**: Le Service de la douleur transitionnelle (STD) de l’Hôpital général de Toronto est un programme multidisciplinaire qui a été mis sur pied au sein même de l’hôpital pour prévenir et prendre en charge la douleur chronique post-chirurgicale et diminuer l’usage d’opioïdes. Cette étude clinique axée sur les pratiques porte sur les résultats préliminaires du programme de psychologie du STD. Ce programme offre une thérapie d’acceptation et d’engagement (ACT) aux patients à risques de douleur post-chirurgicale chronique et d’usage persistant d’opioïdes.

**Méthodes**: Quatre-vingt onze patients ont bénéficié de l’ACT, tandis que deux-cent cinquante-deux patients n’en ont pas bénéficié (groupe sans ACT). Les résultats obtenus ont été comparés pour les patients des deux groupes lors de la première et de la dernière visite d’ACT. Une analyse de variance à deux facteurs (groupe [ACT - sans ACT] x moment [première, dernière visite]) a été effectuée pour la douleur, l’interférence de la douleur, la sensibilité au traumatisme de la douleur, la catastrophisation de la douleur, l’anxiété, la dépression et l’usage d’opioïdes.

**Résultats**: Les résultats suggèrent que les patients référés à l’ACT étaient plus susceptibles de souffrir d’un problème de santé mentale avant l’opération chirurgicale (*p* < 0,001) et présentaient un plus grand usage d’opioïdes (*p* < 0,001) au moment de la première visite post-chirurgicale. De plus, ils manifestaient une plus grande prédisposition a la douleur en lien avec un sensibilité au traumatisme (*p* < 0,05) et à l’anxiété (*p* < 0,05) comparativement au groupe sans ACT. Une diminution de la douleur, de l’interférence de la douleur, de la catastrophisation en lien à la douleur et de l’usage d’opioïdes au moment de la dernière visite au STD (*p* < 0,05) a été observée chez les sujets des deux groupes. Une plus grande diminution de l’usage d’opioïdes, de l’interférence de la douleur et de l’humeur dépressive (*p* = 0,001) ont été observées chez le groupe avec ACT à la fin du traitement, ceci comparativement au groupe sans ACT.

**Conclusion**: Les résultats préliminaires suggèrent que l’ACT a été efficace pour réduire l’usage d’opioïdes tout en diminuant l’interférence de la douleur et en améliorant l’humeur des patients.

## Introduction

Public health concerns related to high-dose, long-term opioid use for the treatment of persistent pain have reached a fever pitch. These concerns are fueled by the lack of evidence for the effectiveness of opioids in adequately treating or alleviating many chronic pain conditions,^1^ risks of harmful side effects,^2^ drug diversion and illicit opioid use,^3^ addictions,^4^ and fatal overdoses.^5^ A significant proportion of chronic pain and problematic opioid use issues can be linked to the acute phase after major surgery, when patients are commonly prescribed opioid analgesics to manage their postsurgical pain.^6,7^ Most patients are provided little more than pharmacotherapy for pain after surgery and are generally offered no intervention targeting the psychological vulnerability factors that are associated with a greater probability of pain chronicity, including pain catastrophizing,^8^ sensitivity to pain traumatization,^9^ anxiety and depressive symptoms.^10^ In addition, although behavioral interventions have been shown to reduce the impact of persistent pain in terms of reducing pain interference (i.e., the impact of pain on work, sleep, relationships, enjoyment of life, etc.),^11^ postsurgical patients have not historically been offered these interventions. Furthermore, they are typically offered very little medical or behavioral guidance to support opioid weaning,^12^ given the general expectation that postsurgical pain will resolve and pain medication use will naturally taper in parallel. However, between 5% and 70% of surgery patients develop chronic postsurgical pain (CPSP), depending in part upon the surgical procedure.^13^ Moreover, 3% and 0.4% of previously opioid-naïve patients continue to use opioids 3 months^7^ and 1 year after major surgery, respectively, likely due to persistent postsurgical pain.^6^

Recent U.S. national guidelines for managing postsurgical pain have emphasized that opioids should not constitute a stand-alone treatment for postoperative pain and should be combined with nonpharmacological approaches to prevent long-term opioid use.^12^ Patients taking a selective serotonin reuptake inhibitor (SSRI) or a benzodiazepine prior to surgery to treat depression or anxiety have been identified as having the highest risk of persisting on opioids following major surgery.^6,14^ The combination of postsurgical pain, disability, and continued psychological symptoms can be overwhelming for patients in the early recovery period. Thus, a concerted effort must be made to address depression and anxiety in the months after surgery because they have the potential to amplify pain, pain interference, and opioid use.

The Toronto General Hospital has developed an innovative Transitional Pain Service (TPS) that uses multidisciplinary methods to prevent and manage CPSP.^15^ The TPS provides physician-guided opioid medication management and tapering, as well as opioid-sparing, nonopioid pharmacotherapy (e.g., alpha-2-delta ligands for postsurgical neuropathic pain). The TPS also offers behavioral interventions grounded in Acceptance and Commitment Therapy (ACT) that are tailored to the postsurgical population and address pain education, pain coping, pain interference, and mood and anxiety concerns.^16^

ACT has garnered significant research support as an effective behavioral chronic pain intervention in a relatively short amount of time^17,18^ and has been rated as having strong research support in the treatment of chronic pain by Division 12 of the American Psychological Association.^19^ Systematic review has suggested that ACT is particularly effective in terms of reducing distress and improving functioning, particularly physical functioning, in people living with long-term pain.^20^ Acceptance-based interventions have also been shown to reduce pain intensity^21^; however, there are indications that acceptance-based interventions that focus on mindfulness mediation training (e.g., mindfulness-based stress reduction) may be associated with greater reductions in pain intensity than ACT interventions.^21,22^ This is consistent with the theoretical framework of ACT for the treatment of chronic pain, which does not emphasize reductions in pain intensity per se, because single-minded pursuit of pain relief is considered ineffective and unworkable once pain has become entrenched.^18^ Instead, ACT intervention is focused on fostering committed engagement with valued life activities, while promoting mindfulness and acceptance of difficult private experiences, such as pain. This is consistent with the sometimes controversial perspective that multidisciplinary treatments for chronic pain, including medication management, are most effectively targeted at reducing pain-related distress and disability, rather than reducing pain intensity.^23^ At the TPS, psychology services consist of individual and group sessions of ACT led by a clinical psychologist and supervised trainees who help patients to develop behavioral skills that make pain more manageable and thus support opioid tapering, so that patients can achieve “a balance between the benefits and potential harms of opioids” (p. 2098).^23^

The TPS psychology service provides proactive, timely support in a multidisciplinary setting to outpatients with complex postsurgical pain for up to 6 months after surgery. TPS patients are flagged for presurgical psychosocial or mental health problems, chronic opioid use, and/or pre-existing chronic pain in order to ensure that an intensive intervention is provided to patients who are at the highest risk for developing CPSP and persistent high-dose opioid use.^15^ To our knowledge, this is the first hospital-integrated, comprehensive, long-term postsurgical pain management program of its kind. Thus, little data are available on clinical outcomes of postsurgical patients who receive psychological support to help them manage complex and persistent postsurgical pain, opioid use, and psychosocial symptoms.

We undertook analyses on preliminary data from the TPS based on patients’ usage of psychological services, specifically examining changes in pain, pain interference, sensitivity to pain traumatization, pain catastrophizing, anxiety and depressive symptoms, and opioid use throughout the duration of their TPS treatment. We hypothesized significant decreases in pain, opioid use, pain interference, pain catastrophizing, sensitivity to pain sensitization, and anxiety and depressive symptoms in patients who received psychology services. Given that TPS psychology services were provided to patients with a high risk of developing CPSP and long-term, high-dose opioid use, we also explored pain, psychosocial outcomes, and opioid use in patients who did not receive any psychology treatment to examine the extent of improvement between the two groups.

## Methods

The study was reviewed and approved by the research ethics boards of Toronto General Hospital (University Health Network, Toronto, ON, Canada) and York University (Toronto, ON). All patients provided informed written consent to participate.

### Participants

Data were collected on 382 patients (194 male, 172 female, mean age = 51.96 years, SD = 14.4, range = 19–83 years) attending the TPS clinic between April 2014 and November 2016. Patients were referred to the TPS by one of three care pathways: (1) referred and followed prior to their surgery date due to risk factors for postsurgical pain and followed as TPS outpatients after surgery (*n* = 89, 23.30%); (2) referred by the Acute Pain Service (APS) after surgery and followed by the TPS as surgical inpatients, with continued care as outpatients after hospital discharge (*n* = 155, 40.6%); or (3) referred by surgeons or primary care physicians to the TPS outpatient clinic when pain persisted after postsurgical hospital discharge (*n* = 118, 30.89%). Two postsurgical patients self-referred to the TPS. Overall, 364 patients attended the TPS clinic for post–hospital discharge visits after major surgery (cardiac 11.1%; thoracic 23.2%; ear, nose, and throat 13.1%; general 19.9%; transplant 8.1%; vascular 8.4%; other 10.4%). Patients who attended the TPS and received treatment for chronic pain unrelated to major surgery were excluded from this study (*n* = 18).

Patients were referred to the TPS clinical psychologist by a TPS physician or an APS nurse. Criteria for receiving psychology services included report of moderate-to-severe acute postsurgical pain or persistent postsurgical pain (≥4 on the 0–10 numeric rating scale for pain intensity) or any one or more of the following: (1) pre-existing chronic pain; (2) physician- or nurse-assessed clinical depression, anxiety, and/or problematic opioid use; (3) physician or nurse clinical judgment that the patient was demonstrating marked difficulty coping with postsurgical pain, compared to the average postsurgical patient who has undergone an equivalent procedure; or 4) daily opioid use higher than expected and/or persisting at high doses for longer than expected in the postsurgical recovery period, with possible psychological barriers to opioid tapering. Because this was not a controlled trial, referral criteria were not strictly operationalized but were based on clinical judgment.

### Study groups

Patients who received one or more ACT intervention sessions after postsurgical hospital discharge (*n* = 91) were classified as belonging to the ACT group. Postsurgical patients who did not receive any psychology services were classified as belonging to the no ACT group (*n* = 252). Twenty-one patients were assessed by psychology services but did not elect to participate in ACT sessions. These 21 patients were excluded from the analyses in order to avoid confounding the study groups.

### Psychology treatment—ACT intervention

All psychology sessions were provided by a registered clinical psychologist (AZW) with 10 years of experience using ACT and/or one of two supervised PhD-level clinical psychology graduate students (MAA, LCB). Treatment was primarily delivered in one-on-one sessions, with a minority of patients attending a pilot series of group sessions. All sessions followed an ACT treatment protocol designed to reduce struggle against difficult inner experiences (such as pain) while, at the same time, fostering long-term patterns of behavior aimed at building a rich and rewarding life.^24,25^ The ACT approach to behavioral pain management was taught to patients using a visual diagram called the ACT matrix (Figure 1a).^24^ Patients were guided in using the matrix to observe their internal reactions to pain; practice awareness of avoidance behaviors (“away moves”) that aim to control pain, including taking pain medication; and engage in activities (represented in the “toward moves” quadrant) that nurtured their connections with loved ones and fulfilled their values (e.g. better health, strong family ties, enjoyment of life; Figure 1b). During sessions, patients were guided through brief mindfulness practices that taught them a new way of cultivating present moment awareness and non-reactivity to pain sensations, while observing pain-related emotions and thoughts.^26–28^ They were encouraged to practice mindfulness at home for 10 minutes per day. Each session lasted ~45 minutes and consisted of a combination of ACT matrix and mindfulness practices designed to provide new skills for coping with persistent pain as patients tapered their opioids and worked toward their desired level of activity.

**Figure 1a.**
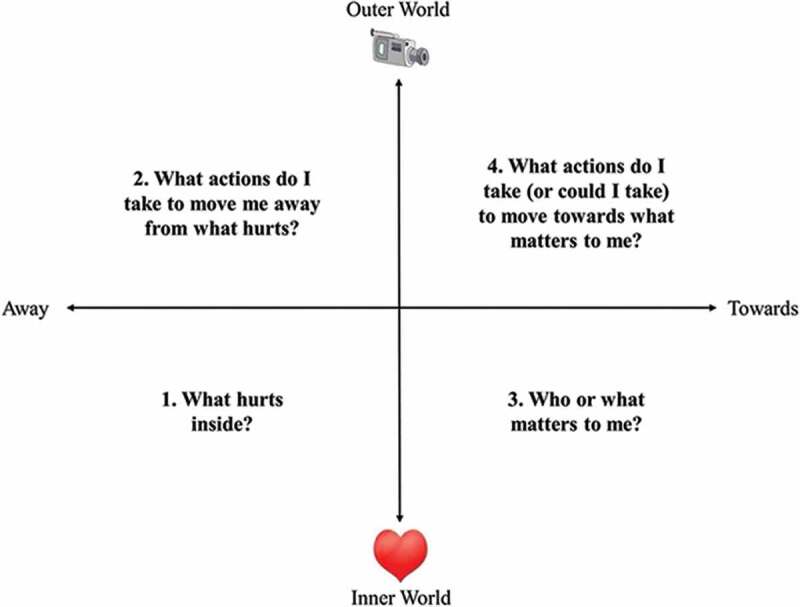
ACT group patients are supported in taking a new perspective on their pain and their responses to pain and developing awareness of their behavioral choices, in order to build a life of meaning and purpose.

**Figure 1b.**
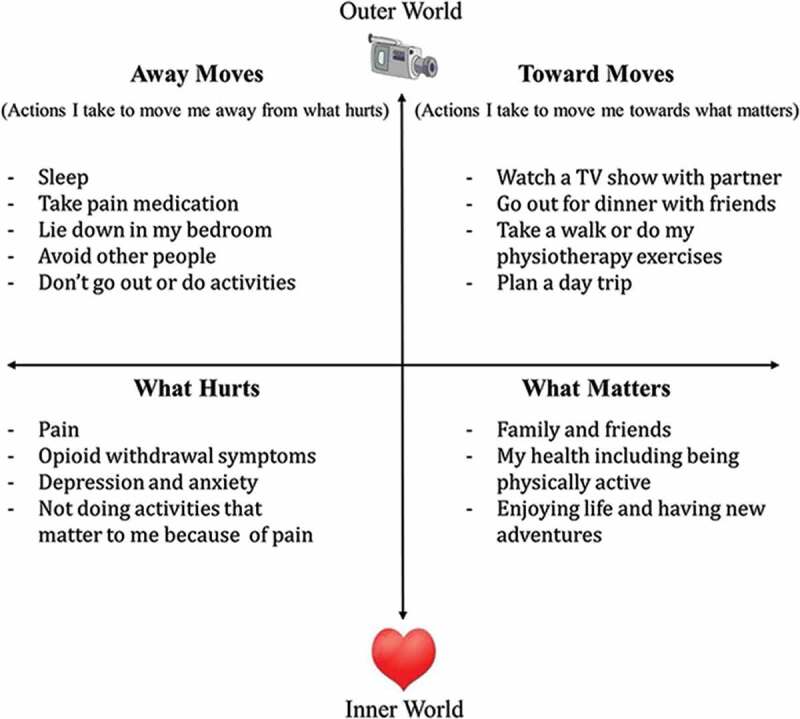
Depiction of an ACT matrix visual diagram for a TPS patient in the ACT group. Lower half represents the inner, private world of the patient, with the right quadrant encapsulating values and important relationships (“what matters”) and left quadrant representing pain and suffering (“what hurts”). Upper half represents the observable world of actions, with the right quadrant signifying meaningful approach behaviors that move patients toward what matters to them (“toward moves”) and left quadrant indicating avoidance behaviors that move patients away from pain and suffering (“away moves”).

### Measures

Demographic and clinical health information (history of diagnosed and treated mental health disorders, medication use, number of morbidities, history of chronic pain) was collected by nurses in the surgical pre-admission clinic as part of routine clinical care by (1) use of a standardized pre-admission interview and (2) review of the patient’s hospital-based electronic medical record. Health information from the pre-admission visit note was later abstracted from the electronic medical record by the research team. Patients were asked to complete questionnaire packages at pre-surgery, post-surgery in-hospital, and post-hospital discharge outpatient TPS visits. For the purposes of analyses, we used the first and last post-hospital discharge outpatient questionnaire data. The questionnaire package included the following inventories.

#### The Brief Pain Inventory–Short Form

The Brief Pain Inventory–Short Form (BPI-SF)^29^ is a 16-item scale that assesses current, least, average, and worst pain intensity using a numeric rating scale (NRS) of 0 (*no pain*) to 10 (*pain as bad as you can imagine*). It contains seven pain interference subscales that measure how pain interferes with various daily activities including general activity, walking, work, mood, enjoyment of life, relations with others, and sleep. Patients rate each aspect of pain interference using an 11-point NRS (0 = *does not interfere*; 10 = *interferes completely*). Pain interference is measured as a mean of the seven subscales, with higher scores meaning that pain interferes to a higher degree in the patient’s functioning. The pain interference subscale has good internal consistency (α = 0.80) in clinical samples.^30^ Internal consistency of the BPI-SF pain interference subscale for the present study was 0.94.

#### Sensitivity to Pain Traumatization Scale–12

The Sensitivity to Pain Traumatization Scale–12 (SPTS-12)^31^ is a 12-item, self-report scale that measures vulnerability to developing anxiety-related cognitive, emotional, and behavioral reactions to pain that resemble symptoms of a traumatic stress reaction (sample items: “When I’m in pain, things don’t feel real”; “Pain sensations terrify me”; “When I’m in pain, everything I see or do reminds me of the pain”; “When I’m in pain, I feel distant from people even when I’m talking to them”). Each item is rated on a five-point rating scale using the following response options: 0 = *not at all true*; 1 = *slightly true*; 2 = *somewhat true*; 3 = *very true*; and 4 = *entirely true*. Total scores (sum of 12 items) range from 0 to 48, with higher scores indicating higher levels of sensitivity to pain traumatization. The SPTS-12 has a one-factor structure, good convergent and divergent validity, and excellent internal consistency (0.84–0.92) in community and clinical samples.^31,32^ The SPTS-12 predicts pain intensity and pain interference post–hospital discharge in patients who have undergone major surgery.^33^ Internal consistency of the SPTS-12 for the present study was 0.92.

#### Pain Catastrophizing Scale

The Pain Catastrophizing Scale (PCS)^34^ is a 13-item scale that measures catastrophic thinking in relation to pain. Each item is rated on a five-point rating scale using the following response options: 0 = *not at all*; 1 = *to a slight degree*; 2 = *to a moderate degree*; 3 = *to a great degree*; and 4 = *all the time*. The PCS yields a total score and three subscale scores assessing rumination (e.g., “I can’t seem to keep it out of my mind”), magnification (e.g., “I wonder whether something serious may happen”), and helplessness (e.g., “There’s nothing I can do to reduce the intensity of the pain”). The PCS has demonstrated good convergent and divergent validity and excellent internal consistency (α = 0.95) in a sample of chronic pain patients.^35^ Internal consistency of the PCS for the present study was 0.96.

#### Hospital Anxiety and Depression Scale

The Hospital Anxiety and Depression Scale (HADS)^36^ is a widely used 14-item scale that assesses symptoms of anxiety (HADS-A; seven items) and depression (HADS-D; seven items) among medical inpatients, outpatients, and in the general population. For each item, the patient is asked to select from among five possible responses (*very often, quite often, not very often, not at all*) the one that best describes how they have been feeling over the past week. Suggested clinical cutoffs for the HADS are 8–10 for mild, 11–15 for moderate, and ≥16 for severe depression or anxiety.^37^ The HADS has demonstrated good internal consistency in clinical samples (HADS-A; α = 0.76; HADS-D; α = 0.80).^37^ Internal consistencies of HADS-A and HADS-D for the present study were 0.82 and 0.83, respectively.

#### Opioid use

The amount of opioids used in a 24-hour period was recorded in the medical charts by TPS physicians at each patient visit (including deviations from prescribed amounts) and abstracted from the medical record. Opioid use was calculated as total morphine equivalent dose (MED) per 24 hours based upon the scientific literature^38,39^ and the conversion methods used by physicians and nurses at UHN. Values obtained at the first and last outpatient TPS visits for all outcomes were included in the analyses to examine changes over the duration of TPS involvement.

### Data analyses

Data were transformed when assumptions of normality were not met, as was the case for MED (log transformed), depressive symptoms (log transformed), and pain catastrophizing (square root transformed).

Age was analyzed using one-way analysis of variance (ANOVA). Preoperative MED, number of weeks of TPS attendance, number of medical visits, and number of comorbidities were analyzed using a Kruskal-Wallis H test with group (ACT, no ACT) as the independent variable. Sex, preoperative chronic pain, preoperative opioid use, preoperative mental health conditions, and preoperative mental health medications were analyzed using Pearson’s Chi-squared tests of likelihood followed by inspection of standardized residuals within cells to determine the pattern of significance among cells. Separate Chi-squared tests were used to examine differences in TPS discharge opioid status (on opioids, opioid-free), between ACT and no ACT groups according to preoperative opioid status (opioid-naïve, opioid users). Clinical outcomes were analyzed by two-way ANOVA using group (ACT, no ACT) and time (first TPS visit, last TPS visit) as factors and current pain, opioid use, pain interference, sensitivity to pain traumatization, pain catastrophizing, anxiety symptoms, and depressive symptoms as dependant variables. Significant interaction effects were followed by tests of simple main effects. Partial eta-squared ($\eta_{p}^{2}$) were used to interpret effect sizes for two-way ANOVAs (small = 0.01, medium = 0.06, large = 0.14).^40^ Wilk’s Lambda values (^) were used to interpret the percent variance in dependent variables not accounted for by independent variables.

## Results

### Group characteristics

Table 1 shows the demographic and clinical variables for the two groups of TPS participants. The ACT group received a mean of 4.90 (SD = 6.46) sessions (median = 2, range 1–33). Eighty patients received one-on-one sessions, eight received both one-on-one and group sessions, and three received only group sessions. ANOVA revealed a significant main effect of group for age, F_(1, 326)_ = 8.08, *P* = 0.005, showing that the ACT group was significantly younger than the no ACT group. Chi-square test indicated a significant association between group and preoperative mental health condition, χ^2^(1) = 17.38, *P* < 0.001: Participants in the ACT group were significantly more likely to have reported a mental health problem at their pre-admission visit (e.g., a mood or anxiety disorder, posttraumatic stress disorder, etc.) than those in the no ACT group. Fifty percent of patients in the ACT group had been taking medications such as SSRIs and anxiolytics preoperatively for mental health conditions compared to 28.5% in the no ACT group. The Kruskal-Wallis H test showed that there was a significant difference in duration (weeks) of TPS involvement between groups, χ^2^(1) = 40.69, *P* < 0.001 (ACT: Q1 = 5.0, median = 17.5, Q3 = 30.75; no ACT: Q1 = 1.0, median = 2.0, Q3 = 11.57), and number of medical visits, χ^2^(1) = 51.02, *P* < .001 (ACT: Q1 = 3, median = 7, Q3 = 10.50; no ACT: Q1 = 1.0, median = 2.0, Q3 = 3.0), with the ACT group having greater weeks of involvement and number of medical visits with the TPS. Sex, number of comorbidities, preoperative chronic pain condition, preoperative mental health medications, preoperative opioid use, and preoperative MED did not differ significantly between the groups. Data on numbers of ACT and no ACT group patients using opioids at TPS discharge according to preoperative opioid use are presented in Table 2. Chi-squared test indicated a significant association between TPS discharge opioid status and treatment groups, χ^2^(1) = 4.37, *P* < 0.05, for preoperatively opioid-naïve patients: A greater proportion of preoperative opioid-naïve patients in the ACT group were completely weaned off opioids (65.4%) compared to the no ACT group (41.8%; Table 2).

**Table 1.** Demographic and clinical variables for the two groups of TPS participants.^a^

	ACT (*n* = 91)		No ACT (*n* = 252)	
Characteristic	*n*	Mean (SD) or %	*n*	Mean (SD) or %
Age (years)	89	48.36 (13.76)	238	53.38 (14.37)
Gender				
Male	43	48.39	132	44.50
Female	46	51.70	106	55.50
TPS attendance (weeks)^b^	84	22.09 (19.96)	198	10.40 (17.80)
Medical visits	85	8.11 (7.32)	199	3.42 (4.87)
Psychology visits	91	4.90 (6.46)	—	—
Number of morbidities^c^				
None	1	1.12	3	1.62
1–3	41	46.07	103	55.68
4–6	21	23.40	61	32.97
7–9	9	10.11	16	8.65
≥10	2	2.24	2	1.08
Preoperative chronic pain				
Yes	54	59.30	121	48.00
No	20	22.00	69	27.40
N/A^d^	17	18.70	62	24.60
Preoperative mental health condition				
Yes	33	41.30	33	17.40
No	47	58.80	157	82.60
Preoperative mental health medications				
Antidepressants	11	12.50	16	7.30
Anticonvulsants	6	6.80	1	0.50
Anxiolytics	13	14.80	20	9.0
SNRI	8	9.10	10	4.50
SSRI	6	6.80	16	7.20
Preoperative opioid use				
Yes	52	58.40	113	47.10
No	37	41.60	127	52.90
Preoperative MED^e^	52	95.98 (121.04)	113	95.63 (128.09)

^a^Percentages are valid percentages.

^b^Time (in weeks) in between first and last TPS outpatient visits.

^c^Refers to any chronic health conditions including preexisting chronic pain.

^d^Preoperative chronic pain status was not available for 86% of patients referred to the TPS after surgery.

^e^MED 24 h presurgery.

TPS = Transitional Pain Service; ACT = acceptance and commitment therapy; N/A = data not available; SNRI = serotonin–norepinephrine reuptake inhibitors; SSRI = selective serotonin reuptake inhibitors; MED = morphine equivalent dosage.

**Table 2.** Number (%) of patients on opioids at TPS discharge in ACT and no ACT groups according to preoperative opioid use.^a^

	ACT (*n* = 91)	No ACT (*n* = 252)
Preoperative opioid users		
TPS discharge opioid-free	11 (22.90)	17 (20.20)
TPS discharge on opioids	37 (77.10)	67 (79.80)
Preoperative opioid-naïve		
TPS discharge opioid-free	17 (65.40)	33 (41.80)
TPS discharge on opioids	9 (34.60)	46 (58.20)

^a^Percentages are valid percentages based on available data.

TPS = Transitional Pain Service; ACT = Acceptance and Commitment Therapy.

### Pain

ANOVA revealed a significant main effect of time, F_(1, 218)_ = 23.10, *P* < 0.001, $\eta_{p}^{2}$ = 0.10, for pain intensity which decreased from the first to last TPS visit (Table 3). The ACT group showed a 17% mean pain score reduction, whereas the no ACT group showed an 8% reduction.

**Table 3.** Mean (SD) values for pain-related variables, mental health variables, and opioid use in the ACT and no ACT groups at the first and last TPS visits.

	ACT (*n* = 91)			No ACT (*n* = 252)		
Outcome	*n*	First visit	Last visit	*n*	First visit	Last visit
Pain intensity^a^	61	5.64 (2.33)	4.69 (2.67)	159	4.93 (2.48)	4.50 (2.55)
Pain interference^b^	58	6.26 (2.39)	4.79 (2.78)	147	5.81 (2.36)	5.09 (2.60)
Sensitivity to pain traumatization^c^	37	22.92 (12.02)	22.81 (12.60)	101	18.37 (11.76)	17.65 (11.62)
Pain catastrophizing^d^	41	26.81 (14.82)	23.28 (15.67)	112	22.61 (14.99)	20.02 (14.48)
Anxiety symptoms^e^	59	10.25 (4.96)	9.88 (5.25)	142	8.65 (4.61)	8.13 (4.65)
Depressive symptoms^e^	59	10.00 (5.27)	8.67 (4.96)	143	9.00 (4.92)	8.66 (4.94)
Morphine equivalent dosage^f^	82	129.18 (146.56)	76.53 (168.86)	193	78.94 (105.13)	57.84 (85.58)

^a^Brief Pain Inventory Current Pain subscale (numeric rating 0–10).

^b^Brief Pain Inventory Pain Interference subscale mean.

^c^Sensitivity to Pain Traumatization Scale-12 total score.

^d^Pain Catastrophizing Scale total score.

^e^Hospital Anxiety and Depression Scale, Anxiety or Depression subscale score.

^f^Morphine equivalent dosage obtained from patient medical charts.

ACT = Acceptance and Commitment Therapy; TPS = Transitional Pain Service.

### Pain interference

**Figure 2.**
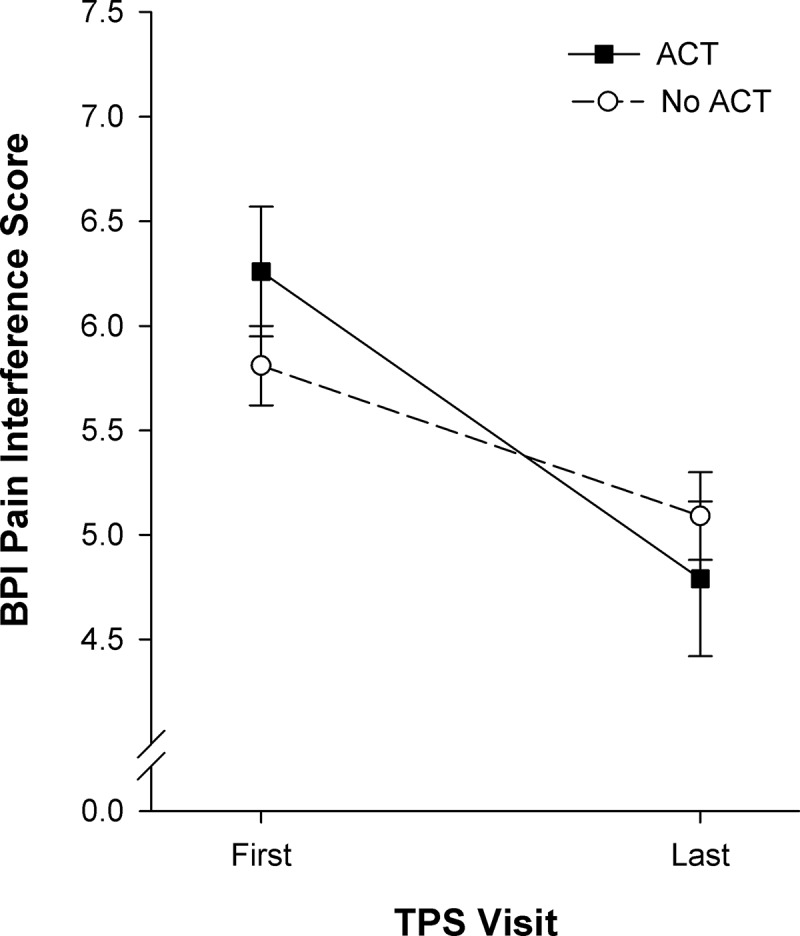
BPI pain interference scores (mean ± standard error of the mean) shown for the two groups of patients at the first and last TPS visits after hospital discharge. The ACT group (*n* = 53) showed greater significant reductions in pain interference scores (*P* < 0.001, effect size $\eta_{p}^{2}$ = 0.14) compared to the no ACT group (*n* = 147, *P* < 0.001, effect size $\eta_{p}^{2}$ = 0.09).

ANOVA revealed a significant main effect of time, F_(1, 203)_ = 52.74, *P* < 0.001, $\eta_{p}^{2}$ = 0.21, and a significant Group × Time interaction, F_(1, 203)_ = 6.07, *P* < 0.05, $\eta_{p}^{2}$ = 0.03. Simple main effects of time were significant for the ACT, ˄ = 0.86, F_(1, 203)_ = 32.98, *P* < 0.001, $\eta_{p}^{2}$ = 0.14, and no ACT, ˄ = 0.91, F_(1, 203)_ = 20.34, *P* < 0.001, $\eta_{p}^{2}$ = 0.09, groups, indicating that pain interference decreased from the first to last TPS visit, with greater decreases occurring in the ACT group (Figure 2). Simple main effects of group were not significant at either time point.

### Sensitivity to pain traumatization

ANOVA revealed a significant main effect of group, F_(2, 136)_ = 4.63, *P* < 0.05, $\eta_{p}^{2}$ = 0.03, indicating that the ACT group had significantly higher sensitivity to pain traumatization scores than the no ACT group at both time points (Table 3).

### Pain catastrophizing

ANOVA revealed a significant main effect of time, F_(1, 161)_ = 13.18, *P* < 0.001, $\eta_{p}^{2}$ = 0.08. All patients showed reductions in pain catastrophizing from the first to last TPS visit (Table 3).

### HADS–Anxiety

ANOVA revealed a significant main effect of group, F_(1, 199)_ = 5.52, *P* < 0.05, $\eta_{p}^{2}$ = 0.03, and time, F_(1, 199)_ = 5.13, *P* < 0.05, $\eta_{p}^{2}$ = 0.03. Pairwise comparisons between group means showed that the ACT group had significantly higher anxiety scores than no ACT group overall. All patients showed reductions in anxiety symptoms from first to last TPS visit (Table 3).

### HADS–Depression

**Figure 3.**
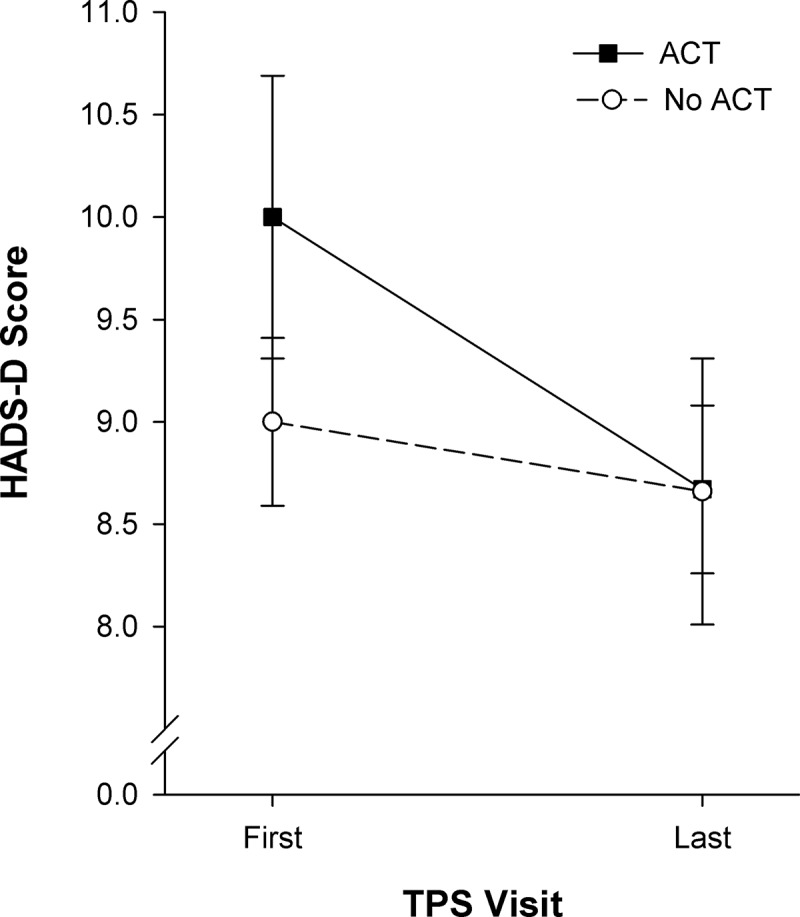
HADS–depression scores (mean ± standard error of the mean) shown for the two groups of patients at the first and last TPS visits after hospital discharge. Statistically significant interaction showed that the ACT group (*n* = 59, *P* = 0.001, $\eta_{p}^{2}$ = 0.05) had significant reductions in depressive symptoms. Depressive symptom scores: 8–10 mild; 11–15 moderate; >16 severe.

ANOVA revealed a significant main effect of time, F_(1, 200)_ = 12.65, *P* < 0.001, $\eta_{p}^{2}$ = 0.06, and a significant Group × Time interaction, F_(1, 200)_ = 4.07, *P* < 0.05, $\eta_{p}^{2}$ = 0.02. The simple main effect of time was significant for the ACT group, ˄ = 0.95, F_(1, 200)_ = 10.98, *P* = 0.001, $\eta_{p}^{2}$ = 0.05, showing reductions in depressive symptoms from first to last TPS visit (Figure 3) but not in the no ACT group, ˄ = 0.99, F_(1, 200)_ = 2.03, *P* = 0.16, $\eta_{p}^{2}$ = 0.01. Simple main effects of group were not significant at either time point.

### Opioid use

**Figure 4.**
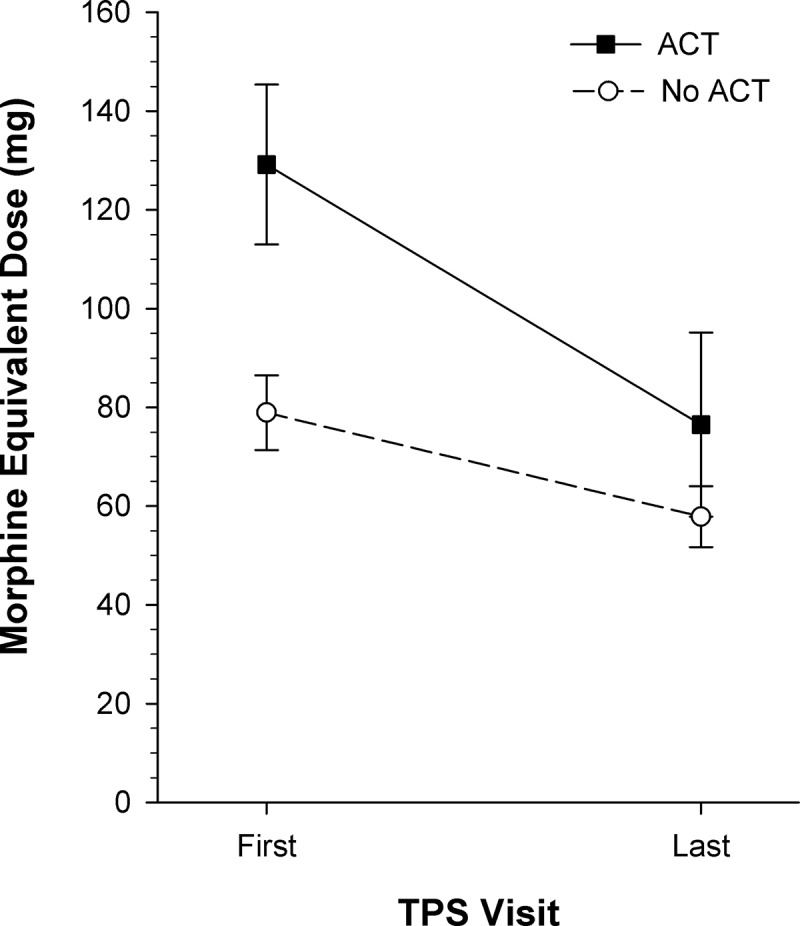
Daily opioid consumption in morphine equivalent dose (mean ± standard error of the mean) shown for the two groups of patients at the first and last TPS visits after hospital discharge. The ACT group had significantly higher opioid use at the first TPS visit than the no ACT group (*P* < 0.001, effect size $\eta_{p}^{2}$ = 0.19) and showed greater significant reductions in opioid use by the last TPS visit (*P* < 0.001). ACT *n* = 82; no ACT *n* = 193.

ANOVA revealed significant main effects of group, F_(1, 274)_ = 5.17, *P* < 0.05, $\eta_{p}^{2}$ = 0.02, and time, F_(1, 274)_ = 74.67, *P* < 0.001, $\eta_{p}^{2}$ = 0.21, and a significant Group × Time interaction, F_(1, 274)_ = 22.35, *P* < 0.001, $\eta_{p}^{2}$ = 0.08. The simple main effect of group was significant at the first TPS visit, F_(2, 274)_ = 18.18, *P* < 0.001, $\eta_{p}^{2}$ = 0.06, indicating that the ACT group had significantly higher MED than the no ACT group at baseline (*P* < 0.001). In contrast, the simple main effect of group was not significant at the last TPS visit, indicating that the ACT group’s MED had reduced to the level of the no ACT group by the last TPS visit (Figure 4). Simple main effects of time were significant for the ACT, ˄ = 0.81, F_(1, 274)_ = 63.89, *P* < 0.001, $\eta_{p}^{2}$ = 0.19, and no ACT, ˄ = 0.96, F_(1, 274)_ = 12.74, *P* > 0.001, $\eta_{p}^{2}$ = 0.04, groups, indicating that both groups showed reductions in MED from first to last TPS visit (Table 3).

## Discussion

This study reports on treatment outcomes for two groups of postsurgical patients treated by a novel, multidisciplinary, transitional pain service in which one group received an ACT-based behavioral pain management intervention and the other did not. The results indicate that both groups improved over the course of the study, showing significant decreases in pain intensity, pain interference, pain catastrophizing, anxiety symptoms, and opioid use by the time of the last TPS visit. Furthermore, the patients who participated in a brief (mean of five sessions) ACT-based pain psychology program in addition to medication management by pain specialist physicians demonstrated significantly greater reductions in opioid use, pain interference, and depressed mood than patients who received physician-guided treatment alone. Differences in time involved with the TPS between ACT and no ACT groups help contextualize the differing needs of complex postsurgical patients. Patients in the ACT group likely had increased needs for follow-up care over time, because their symptoms may have persisted or intensified over a longer period after surgery requiring continued follow-up. Findings of this study should be interpreted with caution due to the inability to attribute symptom improvements among patients in the ACT group mainly to the ACT intervention, because these patients also received significantly more physician-guided care.

It is important to note that these data were obtained in the course of routine practice in the Transitional Pain Service operating in a hospital setting, rather than in the idealized and rigorously controlled conditions of a randomized controlled trial that limit external validity. Accordingly, the present findings should be generalizable to real-world clinical care settings in many hospitals around the globe in which patients have complex pain histories, a variety of mental health conditions, and often a history of presurgical, chronic opioid use. As an investigation of naturalistic clinical care, there were significant differences between the two groups at baseline. Patients who were referred to the ACT-based pain psychology program had significantly higher daily opioid use at their first TPS visit, as well as more anxiety, higher sensitivity to pain traumatization scores, and a greater prevalence of preoperative mental health conditions. Thus, the ACT group was at higher risk of persistent pain, pain-related disability, depression, and long-term, high-dose opioid use.^6,13,41^ However, by the last TPS visit, daily opioid consumption did not differ significantly between the groups, suggesting that the ACT group was able to taper their opioid use by a greater amount. In addition, pain interference decreased to a greater extent in the ACT group. Moreover, only the ACT group showed significant reductions in depressive symptoms by the last TPS visit. Taken together, the results suggest that a higher-risk group of patients who learned the ACT approach to behavioral pain management were able to wean further off opioid medications, while experiencing improvements in mood and less interference from pain in important daily life activities, in comparison to the lower-risk, no ACT group. The results of the present study provide preliminary support for ACT-based intervention targeting patients after surgery who are at risk of CPSP and long-term, high-dose opioid use.

There is a dearth of research investigating the efficacy of psychosocial treatments in reducing opioid use among people living with chronic pain.^42,43^ Meanwhile, concerns continue to rise with regard to the risks of long-term, high-dose opioid use^44^ and dose escalation triggered by postsurgical opioid prescribing practices with little in the way of opioid tapering assistance.^12^ The present study provides preliminary evidence that there is a potentially powerful role for behavioral interventions in supporting opioid tapering among complex pain patients who have undergone major surgery. In addition to the ACT group’s greater mean reductions in daily opioid consumption at TPS discharge, a greater percentage of opioid-naïve ACT group patients tapered their opioid use to zero in comparison to the no ACT group (Table 2). Preventing opioid-naïve patients from becoming chronic opioid users is an important objective in addressing the opioid public health crisis.^44^ To this end, this finding supports the promise of ACT being used in combination with early multimodal medication management to prevent new cases of chronic opioid use after surgery in patients at high risk of persistent postsurgical pain. At the time of TPS referral, patients in the ACT group were taking high doses of opioid medication—well above the 90 mg MED maximum upper limit recommended by the 2017 Canadian guideline for opioids for chronic non-cancer pain.^45^ To change this clinical picture and bring opioid dosing for “legacy patients” in line with current guidelines, empirically supported behavioral protocols are urgently needed to assist patients in tapering their opioid medications,^16^ without a negative impact on functional status or quality of life.

There is a debate among health care professionals who treat people with chronic pain as to the importance of reduced pain intensity as an outcome of multidisciplinary treatment. The present results indicate that both groups showed significant reductions in pain—with the ACT group decreasing by 17% and the no ACT group by 8%—although the magnitude of the difference between groups was not statistically significant. Nonetheless, the mean reductions in pain intensity of the ACT group fell within the 10%–20% range considered to reflect minimally important changes on the 0–10 pain NRS.^46^ Taken together with the ACT group’s reductions in pain interference, this finding is consistent with the literature on ACT interventions that have led to improved functioning in the absence of significant between-group differences in pain intensity.^47–49^

Surgical patients who are highly distressed and anxious are more likely to develop persistent postsurgical pain^13,50^ and to use higher doses of opioids.^6^ The ACT intervention demonstrated differential effects on the various psychological constructs of anxiety and distress assessed across the treatment period. Anxiety and pain catastrophizing decreased in both groups over time but did not decrease more dramatically in the ACT group. Anxiety and catastrophizing may have resolved as time passed, postsurgical healing progressed, and pain decreased in both groups. To date, there has been little investigation on behavioral interventions to reduce postsurgical pain catastrophizing, though a recent uncontrolled pilot study of a single-session pain psychology class specifically tailored for pain catastrophizing has shown large treatment effects in reducing catastrophizing in chronic pain outpatients after 4 weeks.^51^ It is possible that pain catastrophizing reductions would have been greater in the ACT group compared to the no ACT group had the intervention focused more on changing cognitive reactions to postsurgical pain. However, the ACT intervention placed more emphasis on awareness of pain-related cognitions, while reducing their impact on behavior, as reflected in greater reductions in pain interference in the ACT group. In terms of sensitivity to pain traumatization, this was higher in the ACT group at both time points. This is a newer construct in pain psychology, and it may take time to elucidate its importance in predicting clinical outcomes, as well as how best to target it via behavioral interventions. Finally, depression scores improved in the ACT group but not in the no ACT group, despite the fact that many patients are prescribed serotonin–norepinephrine reuptake inhibitors (SNRIs) and atypical antidepressants as adjunct, opioid-sparing medications for postsurgical neuropathic pain, though perhaps not at adequate doses for mood effects. The behavioral activation component of the ACT matrix intervention, which emphasizes engaging in meaningful activities, is designed to alleviate depression at the same time that functioning improves.

## Limitations and future directions

There are several limitations to the present study. First, this research was observational in nature without random assignment to treatment groups; thus, our results may be subject to confounding by treatment indication. However, as described above, the ACT group was more severe with respect to risk factors for postsurgical pain at baseline and thus would be expected to have poorer outcomes—that is, the selection bias observed in the present study would bias our analyses toward the null hypothesis of no difference in outcomes between groups or better outcomes for the no ACT group. The fact that we nonetheless observed decreases in pain, pain interference, pain catastrophizing, anxiety symptoms, and opioid use with outcomes comparable in both the ACT and no ACT group suggests that the strength of effect of the ACT intervention outweighed confounding in the opposite direction. Of particular note, our findings that the ACT group demonstrated greater reductions in depression scores, opioid use, and pain interference than the no ACT group speak to the strength of the ACT intervention.

Another limitation of the present study is that our relatively small sample size precluded full multivariate adjustment and reduced our power to detect underlying treatment effects. If we had sufficient power to control for higher rates of perioperative risk factors for CPSP (such as preexistent chronic pain)^13^ and long-term presurgical opioid use, we may have observed greater effect sizes and additional significant effects of psychological treatment on reducing poor postsurgical outcomes.

The patients in this study underwent a heterogenous set of surgical interventions typical of hospital settings, which may limit the precision of our findings. Different surgical modalities are known to yield different rates of CPSP^13^ and extended opioid use.^6^ Due to small numbers of individuals with each surgery, we lacked sufficient power to run subgroup analyses of differential responses to psychological intervention and TPS treatment overall. Furthermore, heterogeneity of surgical intervention contributes to reduced power to detect potentially real treatment effects and accordingly we are planning randomized controlled trials with intent-to-treat analyses within a given high-risk surgical population.

Finally, all follow-up comparisons were based on group differences at the conclusion of TPS visits, a clinically relevant time point where specialist treatment is discontinued and patients are transitioned back to their primary care physicians. A drawback of this approach is that differences in treatment duration observed between the ACT and no ACT groups represent a confounding variable in this study. Thus, the extent to which the no ACT group would have achieved additional reductions in opioid weaning, pain interference, and depressive symptomology given an equivalent follow-up time is not known. It is also not known whether participation in the ACT treatment protocol increased patient engagement with the service, leading to an increased willingness to attend additional appointments, or whether distressed, yet adherent, patients were more drawn to the option of ACT treatment.

To address these limitations, we aim to run additional analyses with an increased sample size, allowing sufficient power for multivariate adjustment for confounding by indication, follow-up duration, and surgery type subgroups.

## Conclusion

This study reports promising preliminary outcomes from a multidisciplinary transitional pain service that addresses a substantial gap in treatment for patients who have undergone major surgery and are at high risk of developing CPSP and prolonged, high-dose opioid use. The Transitional Pain Service bridges the gap between in-hospital, acute postsurgical pain management and specialized care for chronic pain that is often long delayed and difficult to access. Patients presenting with complex pain problems after surgery receive timely, state-of-the-art, multidisciplinary intervention that provides them with a full repertoire of options for managing their pain in order to reduce reliance on opioid medication. Patients living with pain after surgery understandably want pain relief and need assurance that tapering their opioid medication will not result in more pain, disability, and suffering. The Transitional Pain Service model aims to address these issues in the critical postsurgical period, as part of a new generation of solutions for pain management that address the current public health challenges associated with pain-related high-dose, long-term opioid use.

## References

[1] Chou R, Turner JA, Devine EB, Hansen RN, Sullivan SD, Blazina I, Dana T, Bougatsos C, Deyo RA. The effectiveness and risks of long-term opioid therapy for chronic pain: a systematic review for a National Institutes of Health Pathways to Prevention Workshop. Ann Intern Med. 2015;162:276–286. 25581257 10.7326/M14-2559

[2] Buenaventura R, Rajive Adlaka M, Nalini Sehgal M. Opioid complications and side effects. Pain Physician. 2008;11:S105–S120. 18443635

[3] McDonald DC, Carlson KE. Estimating the prevalence of opioid diversion by “doctor shoppers” in the United States. PLoS One. 2013;8:e69241. 23874923 10.1371/journal.pone.0069241 PMC3714248

[4] Vowles KE, McEntee ML, Julnes PS, Frohe T, Ney JP, van der Goes DN. Rates of opioid misuse, abuse, and addiction in chronic pain: a systematic review and data synthesis. Pain. 2015;156:569–576. 25785523 10.1097/01.j.pain.0000460357.01998.f1

[5] Dhalla IA, Mamdani MM, Sivilotti ML, Kopp A, Qureshi O, Juurlink DN. Prescribing of opioid analgesics and related mortality before and after the introduction of long-acting oxycodone. Can Med Assoc J. 2009;181:891–896. 19969578 10.1503/cmaj.090784 PMC2789126

[6] Clarke H, Soneji N, Ko DT, Yun L, Wijeysundera DN. Rates and risk factors for prolonged opioid use after major surgery: population based cohort study. BMJ. 2014;348:1–10. 10.1136/bmj.g1251 PMC3921439 24519537

[7] Soneji N, Clarke H, Ko DT, Yun L, Wijeysundera DN. Risks of developing persistent opioid use after major surgery. JAMA Surg. 2016;151:1083–1084. 27533746 10.1001/jamasurg.2016.1681

[8] Papaioannou M, Skapinakis P, Damigos D, Mavreas V, Broumas G, Palgimesi A. The role of catastrophizing in the prediction of postoperative pain. Pain Med. 2009;10:1452–1459. 19863742 10.1111/j.1526-4637.2009.00730.x

[9] Kleiman V, Clarke H, Katz J. Sensitivity to pain traumatization: a higher-order factor underlying pain-related anxiety, pain catastrophizing and anxiety sensitivity among patients scheduled for major surgery. Pain Res Manag. 2011;16:169–177. 21766066 10.1155/2011/932590 PMC3198110

[10] Hinrichs‐Rocker A, Schulz K, Järvinen I, Lefering R, Simanski C, Neugebauer EA. Psychosocial predictors and correlates for chronic post‐surgical pain (CPSP)—a systematic review. Eur J Pain. 2009;13:719–730. 18952472 10.1016/j.ejpain.2008.07.015

[11] Morley S. Efficacy and effectiveness of cognitive behaviour therapy for chronic pain: progress and some challenges. Pain. 2011;152(3):S99–S106. 21159433 10.1016/j.pain.2010.10.042

[12] Chou R, Gordon DB, de Leon-Casasola OA, Rosenberg JM, Bickler S, Brennan T, Carter T, Cassidy CL, Chittenden EH, Degenhardt E, et al. Management of postoperative pain: a clinical practice guideline from the American Pain Society, the American Society of Regional Anesthesia and Pain Medicine, and the American Society of Anesthesiologists’ Committee on Regional Anesthesia, Executive Committee, and Administrative Council. J Pain. 2016;17:131–157. 26827847 10.1016/j.jpain.2015.12.008

[13] Katz J, Seltzer Z. Transition from acute to chronic postsurgical pain: risk factors and protective factors. Expert Rev Neurother. 2009;9:723–744. 19402781 10.1586/ern.09.20

[14] Sun EC, Darnall BD, Baker LC, Mackey S. Incidence of and risk factors for chronic opioid use among opioid-naïve patients in the postoperative period. JAMA Intern Med. 2016;176:1286–1293. 27400458 10.1001/jamainternmed.2016.3298 PMC6684468

[15] Katz J, Weinrib A, Fashler SR, Katznelzon R, Shah BR, Ladak SS, Jiang J, Li Q, McMillan K, Santa Mina D, et al. The Toronto General Hospital Transitional Pain Service: development and implementation of a multidisciplinary program to prevent chronic postsurgical pain. J Pain Res. 2015;8:695–702. 26508886 10.2147/JPR.S91924 PMC4610888

[16] Weinrib A, Burns L, Mu A, Azam MA, Ladak SS, McRae K, Katznelson R, Azargive S, Tran C, Katz J, et al. Treatment of complex chronic pain and opioid dependence by a multidisciplinary transitional pain service: utilizing the ACT matrix and buprenorphine and naloxone. J Pain Res. 2017;10 747–755. 28392713 10.2147/JPR.S124566 PMC5376151

[17] McCracken LM, Morley S. The psychological flexibility model: a basis for integration and progress in psychological approaches to chronic pain management. J Pain. 2014;15:221–234. 24581630 10.1016/j.jpain.2013.10.014

[18] Scott W, McCracken LM. Psychological flexibility, acceptance and commitment therapy, and chronic pain. Curr Opin Psychol. 2015;2:91–96.

[19] Division 12 of the American Psychological Association. Acceptance and commitment therapy for chronic pain. Society of Clinical Psychology; 2015; https://www.div12.org/psychological-treatments/disorders/chronic-or-persistent-pain/acceptance-and-commitment-therapy-for-chronic-pain/

[20] Hann KE, McCracken LM. A systematic review of randomized controlled trials of acceptance and commitment therapy for adults with chronic pain: outcome domains, design quality, and efficacy. J Contextual Behav Sci. 2014;3:217–227.

[21] Veehof MM, Oskam M-J, Schreurs KM, Bohlmeijer ET. Acceptance-based interventions for the treatment of chronic pain: a systematic review and meta-analysis. Pain. 2011;152:533–542. 21251756 10.1016/j.pain.2010.11.002

[22] Hughes LS, Clark J, Colclough JA, Dale E, McMillan D. Acceptance and commitment therapy (ACT) for chronic pain: a systematic review and meta-analyses. Clin J Pain. 2017;33:552–568. 27479642 10.1097/AJP.0000000000000425

[23] Ballantyne JC, Sullivan MD. Intensity of chronic pain—the wrong metric? N Engl J Med. 2015;373:2098–2099. 26605926 10.1056/NEJMp1507136

[24] Polk KL, Schoendorff B, Webster M, Olaz FO. The essential guide to the ACT matrix: a step-by-step approach to using the ACT matrix model in clinical practice. Oakland, CA: New Harbinger Publications; 2016.

[25] Hayes SC, Strosahl KD, Wilson KG. Acceptance and commitment therapy: The process and practice of mindful change. New York, NY: Guilford Press; 2011.

[26] Kabat-Zinn J, Lipworth L, Burney R. The clinical use of mindfulness meditation for the self-regulation of chronic pain. J Behav Med. 1985;8:163–190. 3897551 10.1007/BF00845519

[27] Young S. Natural pain relief: how to soothe and dissolve physical pain with mindfulness. Boulder, CO: Sounds True; 2011.

[28] Zeidan F, Grant J, Brown C, McHaffie JG, Coghill RC. Mindfulness meditation-related pain relief: evidence for unique brain mechanisms in the regulation of pain. Neurosci Lett. 2012;520:165–173. 22487846 10.1016/j.neulet.2012.03.082 PMC3580050

[29] Cleeland CS, Ryan K. The Brief Pain Inventory. 1994. [accessed 2016 Mar 1]. https://www.mdanderson.org/documents/Departments-and-Divisions/Symptom-Research/BPI_UserGuide.pdf

[30] Kapstad H, Rokne B, Stavem K. Psychometric properties of the Brief Pain Inventory among patients with osteoarthritis undergoing total hip replacement surgery. Health Qual Life Outcomes. 2010;148:1–8. 10.1186/1477-7525-8-148 PMC3004874 21143926

[31] Katz J, Fashler SR, Wicks C, Pagé MG, Roosen K, Kleiman V, Clarke H. Sensitivity to Pain Traumatization Scale: development, validation, and preliminary findings. J Pain Res. 2017;10:1297–1316 10.2147/JPR.S134133 PMC5459971 28615962

[32] Fashler SR, Montbriand J, Weinrib A, Clarke H, Katz J. Confirmatory factor analysis of the Sensitivity to Pain Traumatization Scale in postsurgical patients seen by the Transitional Pain Service. Research Poster Abstracts of the 2017 Canadian Pain Society Annual Meeting. Can J Pain. 2017;1(1);A93–A94.

[33] Fashler S, Weinrib A, Montbriand J, Clarke H, Katz J. Sensitivity to pain traumatization and perceived injustice predict pain-related outcomes in postsurgical patients seen by the Transitional Pain Service. Paper presented at: 37th Annual Canadian Pain Society Scientific Meeting Annual Meeting; 2016 May 24–27; Vancouver, BC, Canada.

[34] Sullivan MJ, Bishop SR, Pivik J. The Pain Catastrophizing Scale: development and validation. Psychol Assess. 1995;7:524–532.

[35] Osman A, Barrios FX, Gutierrez PM, Kopper BA, Merrifield T, Grittmann L. The Pain Catastrophizing Scale: further psychometric evaluation with adult samples. J Behav Med. 2000;23:351–365. 10984864 10.1023/a:1005548801037

[36] Zigmond AS, Snaith RP. The Hospital Anxiety and Depression Scale. Acta Psychiatr Scand. 1983;67:361–370. 10.1111/j.1600-0447.1983.tb09716.x 6880820

[37] Bjelland I, Dahl AA, Haug TT, Neckelmann D. The validity of the Hospital Anxiety and Depression Scale: an updated literature review. J Psychosom Res. 2002;52:69–77. 11832252 10.1016/s0022-3999(01)00296-3

[38] MD Anderson Cancer Center. Cancer Pain-Adult Medical Algorithm. MD Anderson Cancer Center; 2015 June [accessed 2017 January]. https://www.mdanderson.org/documents/for-physicians/algorithms/clinical-management/clin-management-post-op-pain-web-algorithm.pdf.

[39] Pereira J, Lawlor P, Vigano A, Dorgan M, Bruera E. Equianalgesic dose ratios for opioids: a critical review and proposals for long-term dosing. J Pain Symptom Manage. 2001;22:672–687. 11495714 10.1016/s0885-3924(01)00294-9

[40] Richardson JT. Eta squared and partial eta squared as measures of effect size in educational research. Educ Res Rev. 2011;6:135–147.

[41] Savage SR. Long-term opioid therapy: assessment of consequences and risks. J Pain Symptom Manage. 1996;11:274–286. 8636626 10.1016/0885-3924(95)00202-2

[42] Garland EL, Manusov EG, Froeliger B, Kelly A, Williams JM, Howard MO. Mindfulness-oriented recovery enhancement for chronic pain and prescription opioid misuse: results from an early-stage randomized controlled trial. J Consult Clin Psychol. 2014;82:1–3. 24491075 10.1037/a0035798 PMC4076008

[43] Veilleux JC, Colvin PJ, Anderson J, York C, Heinz AJ. A review of opioid dependence treatment: pharmacological and psychosocial interventions to treat opioid addiction. Clin Psychol Rev. 2010;30:155–166. 19926374 10.1016/j.cpr.2009.10.006

[44] Kolodny A, Courtwright DT, Hwang CS, Kreiner P, Eadie JL, Clark TW, Alexander GC. The prescription opioid and heroin crisis: a public health approach to an epidemic of addiction. Annu Rev Public Health. 2015;36:559–574. 25581144 10.1146/annurev-publhealth-031914-122957

[45] Busse JW, Craigie S, Juurlink DN, Buckley DN, Wang L, Couban RJ, Agoritsas T, Akl EA, Carrasco-Labra A, Cooper L, et al. Guideline for opioid therapy and chronic noncancer pain. CMAJ. 2017;189(18):E659–E666. 10.1503/cmaj.170363 PMC5422149 28483845

[46] Dworkin RH, Turk DC, Wyrwich KW, Beaton D, Cleeland CS, Farrar JT, Haythornthwaite JA, Jensen MP, Kerns RD, Ader DN, et al. Interpreting the clinical importance of treatment outcomes in chronic pain clinical trials: IMMPACT recommendations. J Pain. 2008;9:105–121. 18055266 10.1016/j.jpain.2007.09.005

[47] Buhrman M, Skoglund A, Husell J, Bergström K, Gordh T, Hursti T, Bendelin N, Furmark T, Andersson G. Guided Internet-delivered acceptance and commitment therapy for chronic pain patients: a randomized controlled trial. Behav Res Ther. 2013;51:307–315. 23548250 10.1016/j.brat.2013.02.010

[48] Wetherell JL, Afari N, Rutledge T, Sorrell JT, Stoddard JA, Petkus AJ, Solomon BC, Lehman DH, Liu L, Lang AJ, et al. A randomized, controlled trial of acceptance and commitment therapy and cognitive–behavioral therapy for chronic pain. Pain. 2011;152:2098–2107. 21683527 10.1016/j.pain.2011.05.016

[49] Wicksell RK, Melin L, Lekander M, Olsson GL. Evaluating the effectiveness of exposure and acceptance strategies to improve functioning and quality of life in longstanding pediatric pain—a randomized controlled trial. Pain. 2009;141:248–257. 19108951 10.1016/j.pain.2008.11.006

[50] Macrae W. Chronic post-surgical pain: 10 years on. Br J Anaesth. 2008;101:77–86. 18434337 10.1093/bja/aen099

[51] Darnall BD, Sturgeon JA, Kao M-C, Hah JM, Mackey SC. From catastrophizing to recovery: a pilot study of a single-session treatment for pain catastrophizing. J Pain Res. 2014;7:1–8. 10.2147/JPR.S62329 PMC4008292 24851056

